# New Confidence: Optimizing Benchmark Dose Modeling to Improve Risk Assessment

**DOI:** 10.1289/ehp.122-A138

**Published:** 2014-05-01

**Authors:** Carol Potera

**Affiliations:** Carol Potera, based in Montana, has written for *EHP* since 1996. She also writes for *Microbe*, *Genetic Engineering News*, and the *American Journal of Nursing*.

Benchmark dose (BMD) modeling has been touted as a more accurate method for conducting chemical risk assessments.^1^ In this issue of *EHP*, a team of researchers applied BMD modeling to hundreds of chemicals whose health effects are already known in an effort to optimize the approach.^2^

To calculate the highest safe oral dose of a noncarcinogenic chemical, regulators must first estimate the point-of-departure (POD) dose—that is, the dose below which the chemical does not increase disease risk. This POD is then divided by an uncertainty factor to build in extra safety, resulting in a reference dose. In selecting a POD regulators may use the lower confidence limit of the BMD (a value abbreviated BMDL), or they may use the no-observed-adverse-effect level (NOAEL) obtained from animal experiments.

**Figure:**
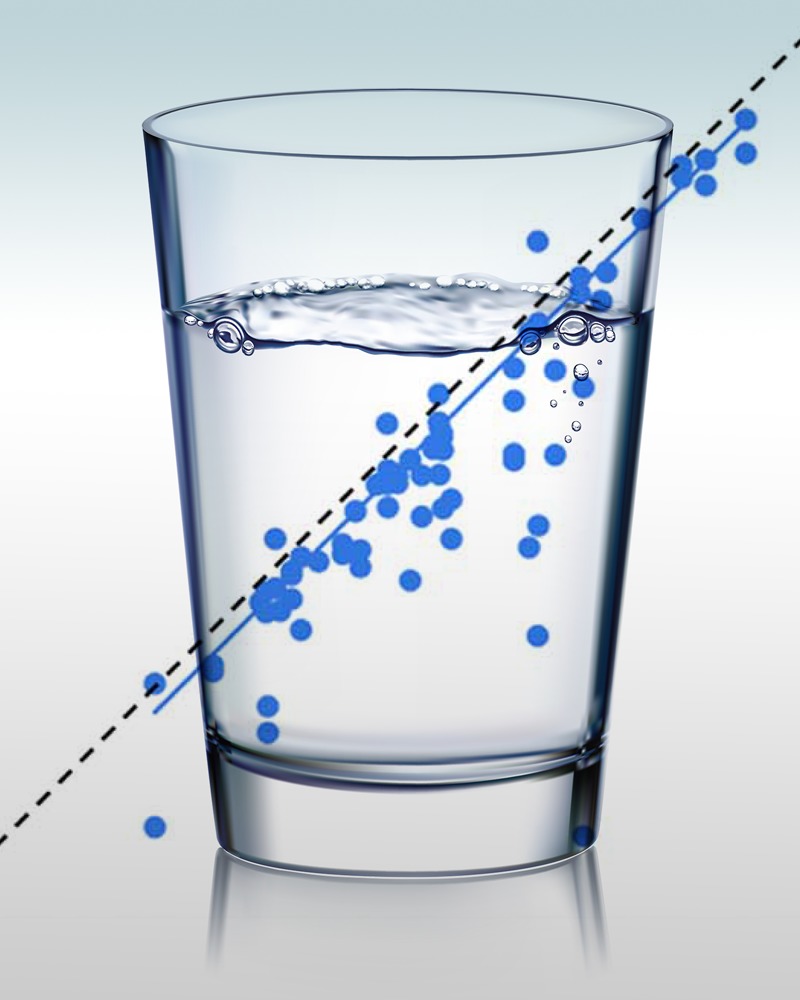
Plotting health outcomes against continuous dose–response data can produce a more accurate point of departure for chemical risk assessment. © Shutterstock; Wignall et al.2; Joseph Tart/Brogan & Partners

Although used since 1954,^3^ NOAEL studies “are not ideal solutions,” says Ivan Rusyn, a toxicologist at the University of North Carolina at Chapel Hill and coauthor of the new study. Often just a few doses of a test chemical are given for varying times to laboratory animals for toxicity testing, and the NOAEL is equal to one of these doses. “This limits NOAEL data,” Rusyn explains, “because the lowest dose may have been arbitrarily set.”

BMD modeling, on the other hand, uses experimental data as the basis for plotting health outcomes against exposure levels along a continuous dose–response curve. (Because of this, BMD analysis is considered inappropriate for certain data sets, such as those with small dosing groups.) Rusyn says a POD derived through BMD modeling can fall to right or left of the NOAEL POD, depending on the data.

In the current study the authors analyzed 880 dose–response data sets for 352 environmental chemicals considered to be human health hazards. BMDLs and NOAELs, they found, gave somewhat similar results, although BMDLs gave slightly lower values, suggesting that toxicity occurs sooner than predicted by NOAELs. “So the BMDL can be more protective than the NOAEL, based on the chemicals we tested,” says Rusyn. However, this result depended on the database.

Researchers typically calculate a BMDL by focusing on data sets for a single chemical at a time. The U.S. Environmental Protection Agency (EPA) has previously used this time-consuming method to derive BMDLs for use in human health risk assessments.^4^ Rather than assess each chemical separately, Rusyn and colleagues processed several hundred data sets for different chemicals, including about 40 chemicals previously evaluated by the EPA. The team followed BMD recommendations described in the EPA’s *Benchmark Dose Technical Guidance* document^5^ and used free BMDS Wizard software.^6^

For those chemicals previously tested by the EPA, the BMDL results correlated well. “It gave us confidence that dose–response analyses can be done faster and more efficiently than scrutinizing one chemical at a time,” Rusyn says.

Overall, the results showed that the standardized approach used by the investigators to calculate the BMDL effectively processed toxicological and epidemiological data for chemicals collected from public sources and compiled in diverse databases. The findings suggested that BMDLs can be calculated in a consistent fashion to improve comparisons across exposures and outcomes. And they also yielded clues about the types of data best suited for BMD modeling.

All these observations trends may help to improve the design of future BMD evaluations, and BMD modeling may help to align high-volume output from toxicity screening with the needs of risk assessors, Rusyn predicts. That’s good, because high-throughput *in vitro* testing is generating huge volumes of new data from hundreds of dose–response data sets on thousands of chemicals.

The researchers “are to be commended for undertaking a comprehensive and insightful analysis of a large database comprised of data-rich chemicals and suggesting a viable approach to standardizing the process for determining BMDLs,” says Daniel Krewski, director of the McLaughlin Centre for Population Health Risk Assessment at the University of Ottawa. This standardized approach to determining a POD in support of risk management decisions should be “more efficient, objective, and transparent than current methods,” Krewski says.

However, the approach does not yield PODs for all data sets considered, notes Krewski. And he points out that expert scientific judgment will still be required to convert a POD to a reference dose.
